# 4K versus 3D total laparoscopic hysterectomy by resident in training: a prospective randomised trial

**DOI:** 10.52054/FVVO.13.3.027

**Published:** 2021-09-24

**Authors:** S Restaino, V Vargiu, A Rosati, M Bruno, G Dinoi, E Cola, R Moroni, G Scambia, F Fanfani

**Affiliations:** Department of Maternal and Child Health, University-Hospital of Udine, P.le S. Maria della, Misericordia n° 15, 33100, Udine, Italy; Division of gynecological oncology, Department of Obstetric and Gynecology, Catholic University of Sacred Heart, L.go A. Gemelli; 00167 Roma (RM), Italy; Scientific Direction, “Fondazione Policlinico Universitario A. Gemelli IRCCS”, Rome, Lazio, Italy; Università Cattolica del Sacro Cuore, Roma, Italy

**Keywords:** laparoscopy, trainee, 4K laparoscopy, 3D laparoscopy

## Abstract

**Background:**

The introduction of ultra-high-definition laparoscopic cameras (4K), by providing stronger monocular depth perception, could challenge the existing 3D technology. There are few available studies on this topic, especially in gynaecological setting.

**Objectives:**

To compare operating times using 3D and 4K vision systems for total laparoscopic hysterectomy performed by surgeons in training.

**Materials and methods:**

In this prospective, single institution, randomised clinical trial (NCT04209036) two laparoscopes utilised were the 0°ULTRA Telescopes with 4K technology and the 0°3D-HD. The surgeons were all trainees and in their last year of residency and who had obtained the certificate of first or second level of the Gynaecological Endoscopic Surgical Education and Assessment (GESEA) programme. Twenty-nine patients with benign uterine pathology were enrolled.

**Main outcome measures:**

Operative time for total laparoscopic hysterectomy.

**Results:**

The 3D vision system did not prove to be superior to the 4K vision system. Operators reported significantly more vision-related side effects when using 3D than 4K. Completing the GESEA training programme was the only factor with a positive and statistically significant impact on the overall time of the procedure, especially when greater dexterity and tissue handling were required.

**Conclusions:**

Neither technology used proved superior to the other, although operators showed a preference for 4K over 3D due to the lower number of visual side effects. Attendance at courses on laparoscopic simulators and training programmes allowed trainees to demonstrate excellent surgical skills.

## Introduction

The use of laparoscopic surgery for the treatment of gynaecological pathologies has steadily increased over the last few decades, both in benign and oncological fields (Aarts et al., 2015; Chapron et al., 2002; Gueli Alletti et al., 2019; Fagotti et al., 2016; Uccella et al. 2019).

The advantages of the laparoscopic approach, compared to laparotomy, are now well known and consist of the reduction of intraoperative blood loss, the reduction of infection and postoperative pain, as well as a shorter hospital stay and better cosmetic results (Murphy et al., 1992; Mais et al., 1996; Yuen and Chang, 1997; Olsson et al., 1996). Conversely, its major limitation is related to the loss of three-dimensional vision with a loss of depth, complicated by the fulcrum effect of laparoscopic instrumentation with inversion of hand movements.

Three-dimensional (3D) vision systems, through depth perception, could theoretically overcome some of these limitations, offering the surgeon a total immersion in the operative field, increasing precision and safety (Vizzielli et al., 2018; Fanfani et al., 2016).

The first 3D laparoscopes were introduced in the 90s, and the first 3D laparoscopic gynaecological procedure was described by Wenzl et al. (1993). Since then, improvements in technology have been made, and most of the side effects, related to the 3D-laparoscopic vision, have been overcome. In fact, the new generation of 3D laparoscopes ensures a high-definition image while reducing the surgeon’s visual fatigue, which was the major reported side effect of the first 3D cameras (Schwab et al., 2017; Sakata et al., 2016). However, conflicting data exists on the advantages of 3D over standard laparoscopic vision systems, and despite its potential advantages, results obtained from prospective clinical studies have been less satisfactory than expected (Koppatz et al., 2020; Zwart et al., 2019; Curtis et al., 2019; Bertolo et al., 2018; Ko et al., 2015; Fanfani et al., 2015; Arezzo et al., 2019). For these reasons, the search for increasingly advanced technologies is still growing steadily today.

The introduction of 4K cameras and the possibility of a magnified vision on 140 cm screens has allowed greater anatomical detail to be obtained thus compensating for the loss of stereoscopic vision, without however the side effects of 3D (Dunstan et al., 2020).

Considering the lack of studies comparing 3D and 4K in gynaecological procedures, we designed a prospective randomised study comparing 3D and 4K vision systems for total laparoscopic hysterectomy performed by surgeons in training. We previously reported the “step by step” technique for laparoscopic hysterectomy with the aim to standardise the surgery (Gueli Alletti et al., 2020), and all the procedures follow these surgical steps.

Secondary aims were to evaluate the possible relationship between the two vision systems (4K and 3D) and the different level of participation of the residents in the training programs on surgical performance.

## Materials and methods

This is a prospective, single institution, randomised clinical trial. Patients were enrolled from April 2020 to September 2020 at Fondazione Policlinico Universitario Agostino Gemelli, IRCCS, in Rome. The study protocol has been approved by the Ethic Committee (protocol number 6499/20 ID 3007) and registered on clinicaltrial.gov platform (NCT04209036).

Informed consent was signed pre-operatively by all women for their data to be collected and analysed for scientific purpose. All data were reported on an anonymous electronic Excel database.

The two laparoscopes utilised were the 10 mm 0° ULTRA Telescopes with 4K technology (Olympus Winter & IBE GMBH, Hamburg - Germany) and the 10 mm 0° 3D laparoscopy high-definition (Olympus Winter & IBE GMBH, Hamburg – Germany).

### Patients

**Figure 1:**
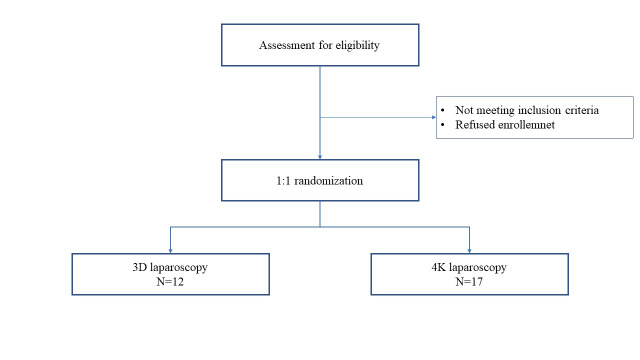
Flow-chart.

All consecutive patients with an indication for total laparoscopic hysterectomy were prospectively enrolled in the study. Inclusion criteria were as follows: pre-operative diagnosis of benign uterine pathology (uterine fibroids, abnormal blood loss, complex hyperplasia with atypia, uterine prolapse) as an indication for total hysterectomy, uterine size ≤ 15 cm at pre-operative evaluation, body mass index (BMI) < 30, American Society of Anesthesiologists (ASA) class ≤ 2 and no diagnosis of pregnancies or pelvic inflammatory disease at the time of the study. Patients with pre-operative suspected neoplastic pathology or not eligible for surgery were excluded from the study. Following a 1:1 randomisation all patients underwent total laparoscopic hysterectomy using 4K or 3D laparoscopes (Figure 1).

### Study participants

All procedures were performed by trainees in their last year of residency, under the supervision of an experienced surgeon (first assistant).

Surgical competency of the trainees involved in the study have been assessed and judged by the Principal Investigator (PI) or by the Co-PI, on the basis of the tissue handling capacity, the competency in identification and dissection of proper anatomical structures and pelvic spaces and appropriate decision making based on intraoperative findings. All operators have experience with the use of the two scopes, both as first or second assistant.

Trainees were further divided into two groups based on their different participation in the training program promoted by the European Academy of Gynaecological Surgery (the Gynaecological Endoscopic Surgical Education and Assessment programme – GESEA programme) (Campo et al., 2016). Trainees who obtained the first level certificate of Bachelor in Endoscopy belonged to the “GESEA-1 group”, while trainees who completed the programme and obtained the certificate and diploma of Minimal Invasive Gynaecological Surgeon belonged to the “GESEA-2 group”.

### GESEA Training Programme

The Gynaecological Endoscopic Surgical Education and Assessment (GESEA) programme is a structured training programme for gynaecological endoscopy, that aims to acquire both theoretical knowledge and practical skills on pelvic simulators, and it is the official certification program of the European Society for Gynaecological Endoscopy (ESGE). It is based on three building blocks:

- Theoretical knowledge which covers laparoscopic anatomy, entry, exposure techniques, energy use, principles and complications of laparoscopy and principles and complications of hysteroscopy.
- Practical skills which implies the execution of 3 tests: the LASTT test (Laparoscopic Skills Training and Testing method), a validated practical test to measure the competence level in basic laparoscopic psychomotor skills; the SUTT test (Suturing and knot tying Training and Testing method), a test consisting of a series of exercises on stitching and knotting to assess the ability of fine and complex motor skills; and the HYSTT test (Hysteroscopic Skills Training and Testing method) to measure the competence level in basic hysteroscopic psychomotor skills.
- Assessment of both theoretical knowledge and practical skills. The first step is the achievement of the GESEA Bachelor Certificate which proves general endoscopic knowledge and acquirement of basic endoscopic psychomotor skills. The second step is to successfully complete the GESEA MIGS (Minimally Invasive Gynecological Surgeon) exam and be awarded the GESEA MIGS Certificate. This certificate indicates that the trainee has mastered the knowledge and more advanced psychomotor skills and has the knowledge and the ability to perform standard procedures in gynaecology.

### Patient characteristics and surgical data

The following pre-intra and post-operative clinical data have been recorded: age at surgery, body mass index (BMI), previous abdominal and uterine surgery, the type of adnexal surgery performed, the use of uterine manipulator, the uterine weight, the estimated blood loss (EBL), the occurrence of intra and post-operative complications (post-operative anaemia and vaginal cuff dehiscence) and the median hospital stay. The Common Terminology Criteria for Adverse Events v3.0 (CTCAE) was used to classify intra-operative complications (CTCAE 0-1 vs CTCAE ≥ 2) and the Extended Clavien Dindo classification of surgical complications has been used for early complications grading definition (Katayama et al. 2016).

All interventions have been recorded and surgical times have been retrieved by the videos. Surgical times have been divided as follows:

- Identification of uterine artery at the origin;
- Coagulation of ovarian pedicles or sealing and section of the mesosalpinx and the utero-ovarian ligament;
- Development of the vescico-uterine septum;
- Colpotomy;
- Suture of the vaginal cuff;
- Total operative time (calculated skin-to-skin).

The operating time of initial adhesion-lysis and final haemostasis were also recorded.

At the end of each surgery, all the operators reported the occurrence of vision-related side effects (eye fatigue, blurred vision, difficulty focusing, and the development of dizziness or nausea) with a score from 0 to 5.

### Statistical analysis

The null hypothesis of the presented study was that the use of 3D laparoscopes for total laparoscopic hysterectomy performed by trainees was associated with a shorter operative time than the same procedure performed using 4K cameras.

Assuming from literature the mean operating time of a TLH is 120 minutes, we estimated that the minimum sample size required to have a 25 % reduction of this time (90 minutes) with an alfa- error=0.05 and beta-error=0.2, was 10 for each arm of the study, rising to 11 per arm assuming a dropout rate of 10%. To have an imbalanced result and to reduce any bias, a randomisation computer list was checked. The two vision systems were compared in terms of operative time, estimated blood loss, incidence of intra or post-operative complications, postoperative pain and days of hospitalisation.

Descriptive statistics have been used to describe the patients and surgical characteristics. The normality of data has been verified via the Kolmogorov-Smirnov test.

Quantitative variables have been described using the following measures: mean and standard deviation (SD) or median, interquartile range (IQR) for not normally distributed variable. Qualitative variables have been summarized with absolute and percentage frequency tables.

Groups were compared using the ANOVA test or the U Mann-Whitney test for continuous variables and the χ^2^ test or Fisher exact test for categorical variables as appropriate.

A two-way ANOVA analysis was conducted to examine the effect that 3D/4K vision systems and/or the achievement of different level during training courses had on the single surgical steps of total laparoscopic hysterectomy and on total operative time.

A p-value < 0.050 have been considered statistically significant (2-tailed test).

Statistical analyses were performed using SPSS version 27.0 (IBM, Armonk, New York, USA).

## Results

At the end of the study period of 6 months, 29 patients had been enrolled: 17 in the 4K group and 12 in the 3D group. Baseline clinico-pathological characteristics of the patients are resumed in Table I. No statistically significant differences among the two study groups were noted in terms of age, BMI, previous abdominal or uterine surgery and indication to surgery. The main indication for surgery was fibroids (58.6% of cases), followed by adenomyosis (24.1% of cases), and atypical endometrial hyperplasia (17.2%).

**Table I:** Baseline patient characteristics.

Variable	All n=29	3D n=12	4K n=17	p-value ‡
Age, years *	53 (47-46)	53 (45-60)	51.5 (48-54.5)	0.679 †
BMI kg/m^2^ *	21.0 (21.0-22.5)	23.6 (21.6-25.8)	23.0 (21.0-27.3)	0.499
Previous abdominal surgery (%)	12 (41.4)	7 (41.2)	5 (41.7)	0.979
Previous uterine surgery (%)	6 (20.7)	3 (17.6)	3 (25.0)	0.669
Indication to surgery (%)				0.556
Fibroids	17 (58.6)	9 (52.6)	8 (66.7)	
Adenomyosis	7 (24.1)	4 (23.5)	3 (25.0)	
Atypical endometrial hyperplasia	5 (17.2)	4 (23.5)	1 (8.3)	

‡ Fisher exact test or Pearsons chi squared test; *median (I-III inter-quartile); † U Mann-Whitney test; BMI: body mass index

Table II states patients’ intra-operative features and the mean time required for each single step of total hysterectomy in both groups (3D and 4K). Of relevance, we did not register differences in the two groups in terms of uterine manipulator utilisation (3D vs 4K: 47.1 vs 41.7%, p=0.774) and the uterine weight (3D vs 4K: 189g vs 240g, p = 0,811). 4K and 3D cases were equally distributed among the trainees’ groups (GESEA 2 group vs GESEA 1 group p=0.774).

**Table II:** Surgical characteristics and operative step times.

Variable	All n=29	3D n=12	3D n=12	
	Surgical characteristics			p-value ^‡^
Adnexal surgery (%)				0.622
Bilateral salpingo-oophorectomy	25 (86.2)	14 (82.4)	11 (91.7)	
Bilateral salpingectomy	4 (13.8)	3 (17.6)	1 (8.3)	
Uterine manipulator (%)				0.774
Yes	16 (55.2)	9 (47.1)	7 (41.7)	
No	13 (44.8)	8 (52.9)	5 (58.3)	
Uterine weight, g*	200 (100-300)	189 (115-305)	240 (100-375)	0.811 ^†^
Operator experience (%)				0.774
GESEA 1	13 (44.8)	8 (47.1)	5 (41.7)	
GESEA 2	16 (55.2)	9 (52.9)	7 (58.3)	
Intraoperative complications (%)	0 (0.0)	0 (0.0)	0 (0.0)	-
EBL, mL*	50 (50-50)	50 (50-50)	50 (50-90)	0.211 ^†^
	Operative step times ^#^			p-value ^§^
Adhesiolysis	6.31 ± 3.04	6.24 ± 3.27	6.60 ± 2.85	0.642
Left uterine artery	7.76 ± 4.18	6.92 ± 3.03	8.93 ± 5.33	0.465
Right uterine artery	7.84 ± 5.78	7.82 ± 5.23	7.87 ± 6.69	0.594
Left adnexal surgery**	3.76 ± 2.21	3.46 ± 2.12	4.16 ± 2.35	0.436
Right adnexal surgery**	4.00 ± 2.32	3.78 ± 2.14	4.30 ± 2.61	0.563
Vesico-uterine fold	8.40 ± 3.94	7.80 ± 2.74	9.27 ± 5.22	0.564
Left uterine pedicle	6.66 ± 5.13	5.90 ± 3.10	7.70 ± 7.13	0.739
Right uterine pedicle	5.77 ± 3.62	6.02 ± 4.16	5.40 ± 2.82	0.825
Colpotomy	9.71 ± 5.82	8.67 ± 5.36	11.18 ± 6.35	0.184
Vaginal cuff suturing	14.84 ± 5.15	15.73 ± 4.86	13.59 ± 5.49	0.156
Haemostasis	7.87 ± 3.69	4.61 ± 2.67	9.44 ± 4.43	0.054
Total operative time	121.55 ± 31.1	124.29 ± 30.722	117.67 ± 32.472	0.593

‡ Fisher exact test or Pearsons chi squared test; * Median (interquartile range); † U Mann-Whitney test; # All variable times are reported in minutes (mean ± standard deviation); § One-way ANOVA test

Mean times were comparable between groups for each surgical step. Total operative time was just slightly lower in the 4K group, but without a statistical weight (3D vs 4K: 124.29 minutes ± 30.722 SD vs 117.67 minutes ± 32.472 SD, p=0.593).

The median post-operative hospital stay was 2 days for both groups, and the number of post- operative complications was extremely low, and none were classified as severe (Grade III-IV of the Clavien-Dindo classification) (Table I S).

Vision-related side effects were higher in the 3D group, following the use of which operators reported greater visual fatigue, blurred vision and difficulty in focusing (p   0.001), while no difference in terms of dizziness or nausea was noted (p=0.062) (Table III).

We then tested the possible interaction between the two vision systems and the different participation of the residents in the training programs on each phase of the intervention and on total operative time, through a two-way ANOVA test (Table IV). The interaction effect between vision system and operator training experience was not statistically significant for any of the examined variables.

An analysis of the main effect for operator training experience was performed, which indicated that the operator main effect was statistically significant for the more difficult steps of total laparoscopic hysterectomy, such as the development of the vesico-uterine fold (operator main effect p = 0.038;vision system effect p = 0.259), the coagulation and cutting of the right uterine pedicle (operator main effect p = 0.005;vision system effect p = 0.711), colpotomy (operator main effect p = 0.021;vision system effect p = 0.173), suture of the vaginal cuff (operator main effect p = 0.001;vision system effect p = 0.313) and on total operative time (operator main effect p = 0.044;vision system effect p = 0.701).

Concerning the main effects of vision systems, no statistically significant differences were reported in any of the total laparoscopic hysterectomy phases.

## Discussion

The laparoscopic 3D vision system did not prove to be superior to the 4K vision system, showing overlapping operative times in all phases of total hysterectomy, and on intra- and post-operative complications. Moreover, operators reported significantly more vision-related side effects when using 3D than 4K.

We did not show any advantage in terms of operative time comparing both the vision systems (3D and 4K) and the GESEA level of the surgeons (Table IV). On the other hand, analysing the two factors individually (3D/4K and GESEA 1/2), completing the GESEA training programme had a positive and statistically significant impact on the overall time of the procedure and in the phases of the intervention where greater dexterity and confidence with tissue handling was required.

Published data on 3D performance versus 2D are extremely disparate and based on hetero-geneous clinical trial and on box-trainer trial.

While studies on simulators (Ko et al., 2015; Spille et al., 2017) and retrospective studies (Usta et al., 2014; Sinha et al., 2018) seem to agree that stereoscopic vision improves surgical performance, especially for more complicated procedures (total laparoscopic hysterectomy in obese patients and for large uteri > 500 g), data from prospective randomised clinical trial did not confirm the same results. In fact, advantages of the 3D system were not found both for simple gynaecological procedures, such as ovarian cystectomy (Lui and Cheung, 2018), or more challenging procedures, such as vaginal cuff closure (Ajao et al., 2020) or radical hys-terectomy for gynaecologic tumors (Fanfani et al., 2015).

A possible explanation of this phenomenon may be related to the fact that, contrary to a vir-tual context, the presence of numerous spatial references within the pelvis could improve the surgeon’s perception of depth.

In addition, it has been shown that about 10% of surgeons cannot perceive stereoscopic depth (Fergo et al., 2016) and many have reported annoying side effects, such as eye fatigue, blurred vision and difficulty focusing (Ko et al., 2015; Lui and Cheung, 2018; Abdelrahman et al., 2018). In accordance with the literature, in our series, participants generally preferred the 4K and reported more visual side effects at the end of interventions performed using the 3D system.

The introduction of ultra-high-definition laparoscopic imaging (“4K”), a two-dimensional technology with four times the number of HD pixels, able to create high resolution images with up to 30 times magnification on a 140 cm screen, potentially optimises surgical performance by providing stronger monocular depth perception signals.

This new technology could actually challenge the existing 3D systems, but few studies are available on the topic, especially in the clinical setting.

On reviewing previous comparisons (2D vs 3D), the studies performed on simulators seem to encourage a better performance with 3D even when compared to 4K, regardless of the individual surgical expertise (Wahba et al., 2020; Harada et al., 2018; Kanaji et al., 2020). On the other hand, when translated into a clinical context, 3D seems to lose its advantages over 4K, probably because the higher definition and high resolution of 4K allowing greater anatomical discrimination, improved dissection and overall surgical performance, without the side effects of 3D (Dunstan et al., 2020).

Our results are consistent with these latest data. Even if the participants of our study were residents, the total operative time did not differ between the two groups, showing that a high-resolution image could improve depth perception in a mono-ocular vision even in the non-experienced surgeon. Moreover, analysing in detail every phase of the intervention, from the simplest to the most complex and busy, the operating times confirmed to be superimposable, strengthening this hypothesis. Furthermore, when asked, participants showed a preference for the 4K system, with which they reported fewer visual side- effects.

The other postulated hypothesis in the literature is that 3D vision systems can facilitate surgeons-in- training, but also on this topic, data is contrasting and mostly based on pelvic-trainer studies (Spille et al. 2017; Ajao et al., 2020).

Indeed, both in our study and in the study by Ajao et al. (2020) 3D did not provide an advantage over monocular vision even by restricting the population of operators to trainees and to complex surgical procedures (vaginal cuff closure and total hysterectomy).

Instead, what we found extremely relevant was the attendance to simulator training programs during the residency. The beneficial impact that simulator-acquired skills have on real surgeries is today undeniable. The possibility of mentoring and learning in a non-stressful environment speeds up the learning curves and self-confidence of trainees, translating into better performance in clinical practice (Wilson et al., 2019; Gala et al., 2013; Larsen et al. 2009; Ahlborg et al., 2013; Lobão et al., 2019).

As demonstrated by our results, thanks to the attendance at courses on laparoscopic simulators provided by our Institution, the trainees in their last year of residency showed excellent surgical skills, performing total laparoscopic hysterectomy in comparable times than those reported in the literature ^1^.

In addition, the standardisation of the surgical technique allowed a comparison of the the single phases of the intervention, regardless of the surgeon who performed the procedure, and allowed evaluation on how the achievement of different levels during the training cours-es resulted in different surgical outcomes.

In detail, trainees with the GESEA 2 level performed better in the more complex phases of the intervention, where the triangulation of the instruments was more uncomfortable (e.g. in the coagulation and section of the right uterine vessels), or where better visuo-motor coordination was required (colpotomy and the suture of the vaginal dome) and at those where greater confidence in tissue handling was needed, as in the development of the vesico-uterine septum.

Furthermore stratifying by groups, the results obtained with 4K have proved to be comparable to those of 3D both in GESEA 1 and GESEA 2 groups, showing that the real advantage, from the point of view of surgical outcomes, was brought by the increased training rather than by the vision system.

This is to date the first randomised-controlled trial in a gynaecological clinical setting to determine the utility of 3D imaging against a 4K system. By choosing residents in their last year of training as the participants in our study, we reduced the bias error that experience could bring. In fact, expert surgeons learn to estimate depth via visual cues, while reducing the potential advantage of new technologies.

One main limitation of the study was the difficulty of measuring surgical performance. However, given the objective nature of time measurement, the standardisation of the surgical technique and video recording, this limitation could be considered acceptable. Furthermore, the group of participants were not completely homogeneous, as some trainees had completed simulator training program while others had only reached the first level, which may have added a bias. In any case, each trainee has been assessed suitable for participation in the study for their laparoscopic surgical skills directly by the PI, therefore even this apparent non-homogeneity of the group can be considered as acceptable.

Thanks to the innovative technology used (3D and 4K), the participants of this study, although still in training, were able to complete a major surgergical procedure with times comparable to those reported in the literature, demonstrating how advancements in technology can speed up the learning curve.

In addition, this study emphasises the need to implement surgical training courses on simulators, showing how residents with the same medical background and experience can significantly improve their surgical performance, dexterity and accuracy thanks to the practice on pelvic trainers.

Starting from these results, and in view of an ever higher surgical efficiency and safety, we firmly believe that the research priorities in minimally invasive gynaecology must be in surgical training and in the introduction of new technologies.

A further step on the road to technological progress could be represented by the introduction of the 8K high-definition imaging system, which through the transmission of true-to-life images should be able to reproduce the sense of the gaze on the original field. This technology could open new possibilities for ever more complex procedures, including more precise nerve and vascular anastomoses, safer surgical resections and of a variety of oncological procedures (Yamashita et al., 2016).

## Conclusion

In conclusion, the surgical successes that we can boast today are undoubtedly largely due to the possibility of using state-of-the-art instrumentation, that thanks to the reproduction of a high-definition 3D vision or 2D images with four times the number of HD pixels, has allowed us to minimise the inherent difficulties of the laparoscopic approach. Neither technology used proved superior to the other, although operators showed a preference for 4K over 3D due to the lower number of visual side effects.

Interestingly, the factor that had the greatest impact on operating times was the completion of surgical training, and in fact, the residents who obtained the MIGS diploma performed better in the most complex steps of the surgery, emphasising how practice and exercise on simulators actually translate into better performance in clinical practice.

We can therefore only adhere to the European– American Joint Recommendation which states that each hospital teaching endoscopic surgery should make available an endoscopic dry lab for training and improving the proficiency of the endoscopic surgery skills of the physician.
